# Supplementary figures and images for: Proteomics and immunocharacterization of Asian mountain pit viper (Ovophis monticola) venom
Source: PLoS One. 2021 Dec 1;16(12):e0260496. doi: 10.1371/journal.pone.0260496 (PMC8635378; doi:10.1371/journal.pone.0260496)

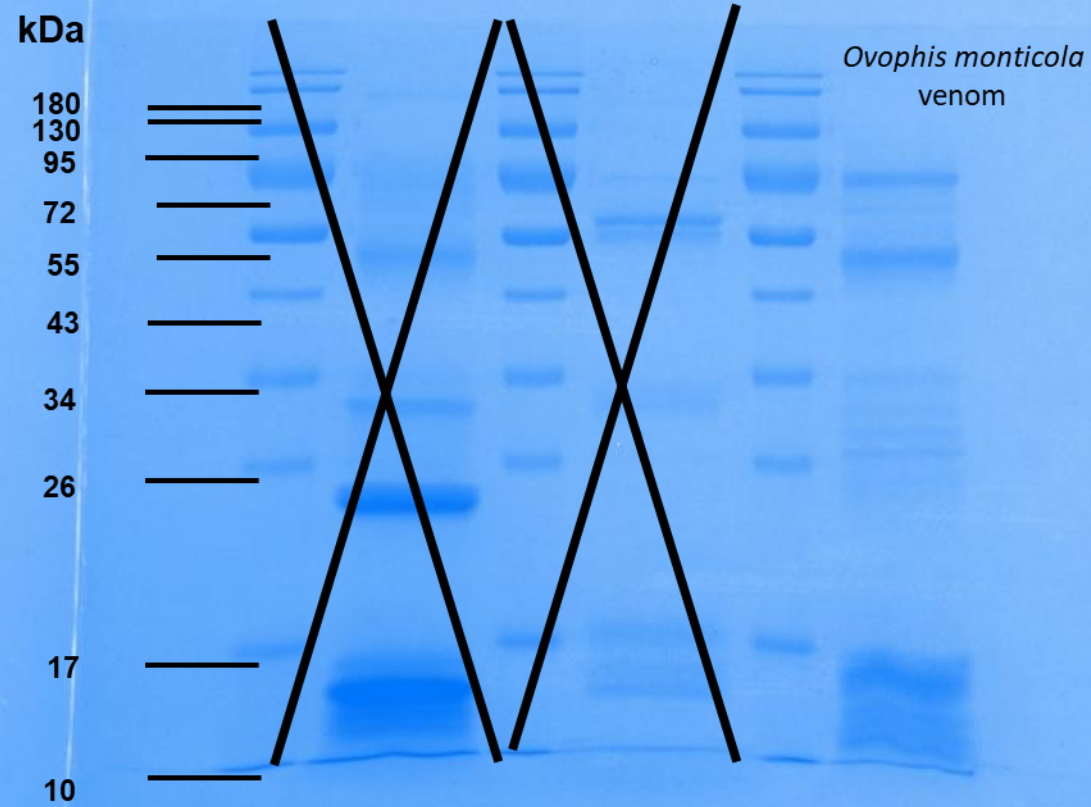

Raw 12% SDS-PAGE gel of Fig.1B

Supplement: S1 Raw image — (PDF) [file pone.0260496.s001.pdf]
