# Supplementary material for: Proteomics and immunocharacterization of Asian mountain pit viper (Ovophis monticola) venom
Source: PLoS One. 2021 Dec 1;16(12):e0260496. doi: 10.1371/journal.pone.0260496 (PMC8635378; doi:10.1371/journal.pone.0260496)
Supplement: S2 Table — Crude venom was subjected to 2DE gel. Proteins were separated in the first dimension in the pH range 3–10. (DOCX) [file pone.0260496.s003.docx]

Table S2: List of proteins in 89 spots in *Ovophis monticola* venom. Crude venom was subjected to 2DE gel. Proteins were separated in the first dimension in the pH range 3–10 and then in the second dimension on a 12% non-linear gradient polyacrylamide gel. The 2DE gel was visualized by silver staining.

| **Spot no.** | **Protein/peptide accession** | **Description [*Organisms*]** | **Prot score** | **Prot mass** | **Prot cover** | **Prot PI** |
| --- | --- | --- | --- | --- | --- | --- |
| 1 | XP_029142019.1 | Zinc metalloproteinase-disintegrin-like atrolysin-A, partial [*Protobothrops mucrosquamatu*s] | 105 | 60272 | 7.5 | 5.53 |
|  | JAS04843.1 | Metalloproteinase type III 2b [*Crotalus horridus*] | 95 | 68297 | 6.1 | 5.54 |
|  | JAS04684.1 | Metalloproteinase type III 1b [*Crotalus adamanteus*] | 83 | 67284 | 6.7 | 4.77 |
|  | AAA03326.1 | Hemorrhagic toxin a (partial)[*Crotalus atrox*] | 82 | 46848 | 5.5 | 5.97 |
|  | GBP06242.1 | Disintegrin and metalloproteinase domain-containing protein 12 [*Eumeta japonica*] | 49 | 199170 | 0.6 | 9.19 |
| 2 | XP_029142019.1 | Zinc metalloproteinase-disintegrin-like atrolysin-A, partial [*Protobothrops mucrosquamatu*s] | 130 | 60272 | 5.6 | 5.53 |
|  | JAS04843.1 | Metalloproteinase type III 2b [*Crotalus horridus*] | 58 | 68297 | 4.3 | 5.54 |
|  | JAS04684.1 | Metalloproteinase type III 1b [*Crotalus adamanteus*] | 48 | 67284 | 2.2 | 4.77 |
| 3 | sp\|Q4VM07.1\|VM3VB_MACLB | Zinc metalloproteinase-disintegrin-like VLAIP-B (Snake venom metalloproteinase) | 82 | 68798 | 2 | 5.93 |
|  | JAS04447.1 | Metalloproteinase type III 7 [*Agkistrodon piscivorus conanti*] | 49 | 68638 | 1.8 | 6.67 |
|  | sp\|P0DM87.1\|VM2_TRIST | Zinc metalloproteinase-disintegrin stejnitin (Snake venom metalloproteinase) | 47 | 54401 | 3.5 | 5.23 |
| 4 | JAS04447.1 | Metalloproteinase type III 7 [Agkistrodon piscivorus conanti] | 86 | 68638 | 1.8 | 6.67 |
|  | sp\|Q4VM07.1\|VM3VB_MACLB | Zinc metalloproteinase-disintegrin-like VLAIP-B | 77 | 68798 | 2 | 5.93 |
| 5 | sp\|Q4VM07.1\|VM3VB_MACLB | Zinc metalloproteinase-disintegrin-like VLAIP-B (Snake venom metalloproteinase) | 94 | 68798 | 2 | 5.93 |
|  | sp\|P0DM87.1\|VM2_TRIST | Zinc metalloproteinase-disintegrin stejnitin (Snake venom metalloproteinase) | 78 | 54401 | 3.5 | 5.23 |
|  | JAS04675.1 | Metalloproteinase type III 5 [*Crotalus adamanteus*] | 62 | 69463 | 1.9 | 7.14 |
|  | JAS04447.1 | Metalloproteinase type III 7 [*Agkistrodon piscivorus conanti*] | 54 | 68638 | 1.8 | 6.67 |
|  | XP_023086434.2 | disintegrin and metalloproteinase domain-containing protein 20-like [*Piliocolobus tephrosceles*] | 46 | 84212 | 5.1 | 6.12 |
| 6 | sp\|Q4VM07.1\|VM3VB_MACLB | Zinc metalloproteinase-disintegrin-like VLAIP-B (Snake venom metalloproteinase) | 90 | 68798 | 2 | 5.93 |
|  | JAS04447.1 | Metalloproteinase type III 7 [*Agkistrodon piscivorus conanti*] | 72 | 68638 | 1.8 | 6.67 |
|  | sp\|P0DM87.1\|VM2_TRIST | Zinc metalloproteinase-disintegrin stejnitin (Snake venom metalloproteinase) | 51 | 54401 | 3.5 | 5.23 |
| 7 | - | Not identified |  |  |  |  |
| 8 | pdb\|1REO\|A | Chain A, Ahplaao | 159 | 55097 | 7.8 | 6.55 |
|  | AAQ16182.1 | L-amino acid oxidase [*Trimeresurus stejnegeri*] | 109 | 58607 | 8.5 | 5.56 |
| 9 | pdb\|1REO\|A | Chain A, Ahplaao | 181 | 55097 | 10.3 | 6.55 |
|  | sp\|A0A024BTN9.1\|OXLA_BOTSC | L-amino acid oxidase Bs29 | 148 | 56341 | 8.2 | 5.79 |
| 10 | sp\|A0A024BTN9.1\|OXLA_BOTSC | L-amino acid oxidase Bs29 | 48 | 56341 | 2.4 | 5.79 |
| 11 | pdb\|1REO\|A | Chain A, Ahplaao | 457 | 55097 | 19.1 | 6.55 |
|  | sp\|A0A024BTN9.1\|OXLA_BOTSC | L-amino acid oxidase Bs29 | 224 | 56341 | 10.4 | 5.79 |
|  | sp\|P0C2D5.2\|OXLA_PROFL | L-amino-acid oxidase (Okinawa Habu apoxin protein-1) | 94 | 3601 | 80 | 4.44 |
|  | sp\|P0C2D6.1\|OXLA_PROMU | L-amino-acid oxidase | 60 | 2929 | 50 | 4.31 |
| 12 | pdb\|1REO\|A | Chain A, Ahplaao | 793 | 55097 | 29.6 | 6.55 |
|  | sp\|A0A024BTN9.1\|OXLA_BOTSC | L-amino acid oxidase Bs29 | 305 | 56341 | 10.6 | 5.79 |
|  | BAP39915.1 | L-amino acid oxidase *[Protobothrops elegans*] | 215 | 57339 | 14.3 | 8.93 |
|  | sp\|P0DI84.1\|OXLA_VIPAA | L-amino-acid oxidase | 214 | 54714 | 12 | 7.71 |
|  | sp\|C0HJE7.2\|OXLA_CRODU | L-amino acid oxidase bordonein-L | 189 | 58882 | 7.8 | 8 |
|  | sp\|Q4F867.2\|OXLA_DABSI | L-amino-acid oxidase | 169 | 46343 | 9.1 | 7.72 |
|  | sp\|X2JCV5.1\|OXLAA_CERCE | L-amino acid oxidase | 130 | 58520 | 5.4 | 6.01 |
|  | sp\|A8QL51.1\|OXLA_BUNMU | L-amino-acid oxidase | 105 | 58774 | 5 | 8.44 |
|  | sp\|P0C2D5.2\|OXLA_PROFL | L-amino-acid oxidase (Okinawa Habu apoxin protein-1) | 103 | 3601 | 80 | 4.44 |
|  | sp\|A0A2U8QPE6.1\|OXLA_MICMP | L-amino acid oxidase | 97 | 57079 | 4.4 | 8.81 |
|  | XP_026523888.1 | titin isoform X41 [*Notechis scutatus*] | 46 | 3637718 | 0.1 | 6.27 |
| 13 | pdb\|1REO\|A | Chain A, Ahplaao | 755 | 55097 |  |  |
|  | JAS04783.1 | L-amino acid oxidase 1b [*Crotalus horridus*] | 311 | 58587 | 11.2 | 6.45 |
|  | sp\|P0DI84.1\|OXLA_VIPAA | L-amino-acid oxidase | 278 | 54714 | 12.2 | 7.71 |
|  | BAP39915.1 | L-amino acid oxidase [*Protobothrops elegans*] | 272 | 57339 | 14.3 | 8.93 |
|  | sp\|A0A024BTN9.1\|OXLA_BOTSC | L-amino acid oxidase Bs29 | 243 | 56341 | 10.4 | 5.79 |
|  | sp\|C0HJE7.2\|OXLA_CRODU | L-amino acid oxidase bordonein-L | 201 | 58882 | 11 | 8 |
|  | JAV01888.1 | BATXLAAO1 [*Bothrops atrox*] | 152 | 56625 | 8.4 | 5.82 |
|  | sp\|Q4F867.2\|OXLA_DABSI | L-amino-acid oxidase | 144 | 46343 | 8.8 | 7.72 |
|  | sp\|P0C2D5.2\|OXLA_PROFL | L-amino-acid oxidase (Okinawa Habu apoxin protein-1) | 132 | 3601 | 80 | 4.44 |
|  | sp\|X2JCV5.1\|OXLAA_CERCE | L-amino acid oxidase | 109 | 58520 | 5.2 | 6.01 |
|  | sp\|A0A2U8QPE6.1\|OXLA_MICMP | L-amino acid oxidase | 101 | 57079 | 4.4 | 8.81 |
| 14 | pdb\|1REO\|A | Chain A, Ahplaao | 582 | 55097 | 21.2 | 6.55 |
|  | sp\|A0A024BTN9.1\|OXLA_BOTSC | L-amino acid oxidase Bs29 | 234 | 56341 | 10.4 | 5.79 |
|  | sp\|P0DI84.1\|OXLA_VIPAA | L-amino-acid oxidase | 190 | 54714 | 8.3 | 7.71 |
|  | sp\|C0HJE7.2\|OXLA_CRODU | L-amino acid oxidase bordonein-L | 176 | 58882 | 7.8 | 8 |
|  | sp\|P0C2D5.2\|OXLA_PROFL | L-amino-acid oxidase (Okinawa Habu apoxin protein-1) | 141 | 3601 | 80 | 4.44 |
|  | JAV01888.1 | BATXLAAO1 [*Bothrops atrox*] | 123 | 56625 | 8.4 | 5.82 |
|  | sp\|A0A2U8QPE6.1\|OXLA_MICMP | L-amino acid oxidase | 94 | 57079 | 4.4 | 8.81 |
| 15 | pdb\|1REO\|A | Chain A, Ahplaao | 397 | 55097 | 16.5 | 6.55 |
|  | sp\|A0A024BTN9.1\|OXLA_BOTSC | L-amino acid oxidase Bs29 | 227 | 56341 | 10.4 | 5.79 |
|  | sp\|A0A2U8QPE6.1\|OXLA_MICMP | L-amino acid oxidase | 99 | 57079 | 4.4 | 8.81 |
|  | XP_026523846.1 | Titin isoform X1 [*Notechis scutatus*] | 53 | 3675875 | 0.2 | 6.29 |
| 16 | pdb\|1REO\|A | Chain A, Ahplaao | 324 | 55097 | 15.4 | 6.55 |
|  | sp\|A0A024BTN9.1\|OXLA_BOTSC | L-amino acid oxidase Bs29 | 161 | 56341 | 9 | 5.79 |
| 17 | pdb\|1REO\|A | Chain A, Ahplaao | 269 | 55097 | 11.1 | 6.55 |
|  | JAV01888.1 | BATXLAAO1 [*Bothrops atrox*] | 117 | 56625 | 6.4 | 5.82 |
| 18 | pdb\|1REO\|A | Chain A, Ahplaao | 717 | 55097 | 27.4 | 6.55 |
|  | AAQ16182.1 | L-amino acid oxidase [*Trimeresurus stejnegeri*] | 374 | 58607 | 12.6 | 5.56 |
|  | sp\|A0A024BTN9.1\|OXLA_BOTSC | L-amino acid oxidase Bs29 | 288 | 56341 | 13.1 | 5.79 |
|  | sp\|P0DI84.1\|OXLA_VIPAA | L-amino-acid oxidase | 200 | 54714 | 10.7 | 7.71 |
|  | JAV01888.1 | BATXLAAO1 [*Bothrops atrox*] | 167 | 56625 | 11 | 5.82 |
|  | sp\|A0A2U8QPE6.1\|OXLA_MICMP | L-amino acid oxidase | 117 | 57079 | 6.8 | 8.81 |
|  | sp\|A8QL51.1\|OXLA_BUNMU | L-amino-acid oxidase | 106 | 58774 | 5 | 8.44 |
|  | sp\|P0C2D5.2\|OXLA_PROFL | L-amino-acid oxidase (Okinawa Habu apoxin protein-1) | 87 | 3601 | 80 | 4.44 |
|  | XP_026523888.1 | Titin isoform X41 [*Notechis scutatus*] | 65 | 3637718 | 0.2 | 6.27 |
| 19 | sp\|A0A024BTN9.1\|OXLA_BOTSC | L-amino acid oxidase Bs29 | 75 | 56341 | 3.8 | 5.79 |
| 20 | BAN82126.1 | Serine protease, partial [*Ovophis okinavensis*] | 128 | 9035 | 30.2 | 8.07 |
|  | JAV51428.1 | Serine proteinase 12a [*Agkistrodon contortrix contortrix*] | 63 | 28885 | 7.4 | 8.23 |
|  | XP_026529526.1 | Microtubule-actin cross-linking factor 1 isoform X1 [*Notechis scutatus*] | 52 | 838459 | 0.6 | 5.38 |
| 21 | sp\|P0C578.1\|VSP2_OVOOK | Thrombin-like enzyme okinaxobin-2 (Fibrinogen-clotting enzyme) | 93 | 2310 | 65 | 4.65 |
|  | JAV51428.1 | Serine proteinase 12a [*Agkistrodon contortrix contortrix*] | 82 | 28885 | 7.4 | 8.23 |
|  | sp\|I2C090.1\|VCO3_OPHHA | Ophiophagus venom factor (Complement C3 homolog) | 49 | 183812 | 0.5 | 6.08 |
|  | XP_026526061.1 | ALK and LTK ligand 1 [*Notechis scutatus*] | 49 | 21543 | 4.7 | 10.61 |
|  | sp\|P85109.1\|VSP1_GLOBR | Thrombin-like enzyme kangshuanmei (Fibrinogen-clotting enzyme) | 44 | 26415 | 9.3 | 8.27 |
|  | JAG68112.1 | Dynamin-binding protein [*Boiga irregularis*] | 43 | 90258 | 2.7 | 6.15 |
| 22 | JAV51428.1 | Serine proteinase 12a [*Agkistrodon contortrix contortrix*] | 71 | 28885 | 7.4 | 8.23 |
|  | BAN82126.1 | serine protease, partial [*Ovophis okinavensis*] | 70 | 9035 | 30.2 | 8.07 |
|  | sp\|E5L0E5.1\|VSPPA_AGKPL | Venom plasminogen activator | 66 | 28060 | 4.3 | 5.78 |
|  | sp\|Q5W958.1\|VSP20_BOTJA | Venom serine proteinase-like HS120 | 47 | 27797 | 4.3 | 8.71 |
| 23 | BAN82126.1 | Serine protease, partial [*Ovophis okinavensis*] | 213 | 9035 | 30.2 | 8.07 |
|  | sp\|Q9PSN3.1\|VSP2_AGKBI | Thrombin-like enzyme bilineobin (Fibrinogen-clotting enzyme/Snake venom serine protease) | 209 | 26461 | 9.8 | 8.6 |
|  | BAN82122.1 | Serine protease, partial [*Ovophis okinavensis*] | 99 | 8080 | 28.8 | 9.92 |
|  | pdb\|2AIP\|A | Chain A, Protein C activator | 96 | 25090 | 8.7 | 8.45 |
|  | sp\|C0HLA2.1\|VSP3_LACMR | Thrombin-like enzyme LmrSP-3 | 69 | 2942 | 50 | 4.1 |
|  | ADI47563.1 | Serine protease, partial [*Echis ocellatus*] | 68 | 27233 | 10.6 | 9.25 |
|  | sp\|P0C5B4.2\|VSPGL_GLOSH | Thrombin-like enzyme gloshedobin(Fibrinogen-clotting enzyme/Snake venom serine protease) | 66 | 28597 | 4.2 | 8.42 |
|  | sp\|Q9DF66.1\|VSP3_PROJR | Snake venom serine protease 3 | 66 | 28007 | 4.3 | 6.49 |
|  | pdb\|1OP0\|A | Chain A, Venom serine proteinase | 62 | 25318 | 5.6 | 5.29 |
| 24 | BAN82126.1 | Serine protease, partial [*Ovophis okinavensis*] | 256 | 9035 | 46.5 | 8.07 |
|  | sp\|Q9PSN3.1\|VSP2_AGKBI | Thrombin-like enzyme bilineobin (Fibrinogen-clotting enzyme) | 207 | 26461 | 9.8 | 8.6 |
|  | BAN82122.1 | Serine protease, partial [*Ovophis okinavensis*] | 117 | 8080 | 28.8 | 9.92 |
|  | pdb\|2AIP\|A | Chain A, Protein C activator | 98 | 25090 | 8.7 | 8.45 |
|  | sp\|C0HLA2.1\|VSP3_LACMR | Thrombin-like enzyme LmrSP-3 (Snake venom serine protease) | 63 | 2942 | 50 | 4.1 |
|  | sp\|P0C5B4.2\|VSPGL_GLOSH | Thrombin-like enzyme gloshedobin (Fibrinogen-clotting enzyme/Snake venom serine protease) | 54 | 28597 | 4.2 | 8.42 |
|  | sp\|Q9DF66.1\|VSP3_PROJR | Snake venom serine protease 3 | 54 | 28007 | 4.3 | 6.49 |
| 25 | BAN82126.1 | Serine protease, partial [*Ovophis okinavensis*] | 158 | 9035 | 30.2 | 8.07 |
|  | sp\|C0HLA1.1\|VSP2_LACMR | Thrombin-like enzyme LmrSP-2 (Snake venom serine protease) | 75 | 3271 | 43.3 | 4.1 |
|  | sp\|C0HLA2.1\|VSP3_LACMR | Thrombin-like enzyme LmrSP-3 (Snake venom serine protease) | 59 | 2942 | 50 | 4.1 |
|  | pdb\|1OP0\|A | Chain A, Venom serine proteinase | 52 | 25318 | 5.6 | 5.29 |
|  | sp\|Q8AY81.1\|VSPST_TRIST | Thrombin-like enzyme stejnobin (Fibrinogen-clotting enzyme/Snake venom serine protease) | 52 | 29309 | 3.5 | 9.11 |
| 26 | JAV51428.1 | Serine proteinase 12a [*Agkistrodon contortrix contortrix*] | 48 | 28885 | 7.4 | 8.23 |
| 27 | XP_029142018.1 | Zinc metalloproteinase-disintegrin jerdonitin [*Protobothrops mucrosquamatus*] | 138 | 58843 | 5.4 | 5.46 |
|  | TSK34762.1 | Disintegrin and metalloproteinase domain-containing protein 12 [*Bagarius yarrelli*] | 53 | 146595 | 0.8 | 8.53 |
|  | XP_032089254.1 | ras GTPase-activating-like protein IQGAP1 [*Thamnophis elegans*] | 47 | 189690 | 2.4 | 6.27 |
| 28 | XP_029142018.1 | Zinc metalloproteinase-disintegrin jerdonitin [*Protobothrops mucrosquamatus*] | 144 | 58843 | 5.4 | 5.46 |
|  | sp\|P0DM87.1\|VM2_TRIST | Zinc metalloproteinase-disintegrin stejnitin | 84 | 54401 | 5.4 | 5.23 |
|  | TSK34762.1 | Disintegrin and metalloproteinase domain-containing protein 12 [*Bagarius yarrelli*] | 53 | 146595 | 0.8 | 8.53 |
| 29 | XP_029142018.1 | Zinc metalloproteinase-disintegrin jerdonitin [*Protobothrops mucrosquamatus*] | 140 | 58843 | 5.4 | 5.46 |
|  | sp\|P0DM87.1\|VM2_TRIST | Zinc metalloproteinase-disintegrin stejnitin (Snake venom metalloproteinase) | 105 | 54401 | 5.4 | 5.23 |
|  | TSK34762.1 | Disintegrin and metalloproteinase domain-containing protein 12 [*Bagarius yarrelli*] | 46 | 215963 | 1 | 7.93 |
|  | ETE65365.1 | putative helicase senataxin, partial [*Ophiophagus hannah*] | 45 | 146595 | 0.8 | 8.53 |
| 30 | XP_029142018.1 | Zinc metalloproteinase-disintegrin jerdonitin [Protobothrops mucrosquamatus] | 145 | 58843 | 5.4 | 5.46 |
|  | sp\|P0DM87.1\|VM2_TRIST | Zinc metalloproteinase-disintegrin stejnitin (Snake venom metalloproteinase) | 108 | 54401 | 5.4 | 5.23 |
| 31 | XP_029142018.1 | Zinc metalloproteinase-disintegrin jerdonitin [*Protobothrops mucrosquamatus*] | 102 | 58843 | 5.4 | 5.46 |
| 32 | BAN82126.1 | Serine protease, partial [*Ovophis okinavensis*] | 143 | 9035 | 30.2 | 8.07 |
|  | sp\|C0HLA1.1\|VSP2_LACMR | Thrombin-like enzyme LmrSP-2 (Snake venom serine protease) | 69 | 3271 | 43.3 | 4.1 |
|  | sp\|C0HLA2.1\|VSP3_LACMR | Thrombin-like enzyme LmrSP-3 (Snake venom serine protease) | 54 | 2942 | 50 | 4.1 |
|  | sp\|Q8AY81.1\|VSPST_TRIST | Thrombin-like enzyme stejnobin (Fibrinogen-clotting enzyme/Snake venom serine protease) | 47 | 29309 | 9.6 | 9.11 |
|  | pdb\|1OP0\|A | Chain A, Venom serine proteinase | 45 | 25318 | 5.6 | 5.29 |
| 33 | - | Not identified |  |  |  |  |
| 34 | JAS05371.1 | Serine proteinase 9d [*Sistrurus miliarius barbouri*] | 85 | 28266 | 12.7 | 7.04 |
|  | sp\|P0DMH6.1\|VSP_BOTFO | Snake venom serine protease | 81 | 1729 | 86.7 | 4.65 |
|  | sp\|Q8AY78.1\|VSP5M_TRIST | Snake venom serine protease 5 | 72 | 28117 | 4.3 | 7.08 |
|  | sp\|Q8AY79.1\|VSPS2_TRIST | Beta-fibrinogenase stejnefibrase-2 (Snake venom serine protease) | 64 | 28010 | 4.3 | 5.37 |
|  | sp\|Q5W958.1\|VSP20_BOTJA | Venom serine proteinase-like HS120 | 54 | 27797 | 4.3 | 8.71 |
|  | sp\|Q71QH7.1\|VSPP_TRIST | Snake venom serine protease PA | 47 | 27933 | 4.3 | 5.66 |
|  | XP_026540424.1 | Inositol hexakisphosphate and diphosphoinositol-pentakisphosphate kinase 1 isoform X1 [*Notechis scutatus*] | 43 | 135476 | 3.2 | 5.51 |
| 35 | sp\|P0DMH6.1\|VSP_BOTFO | Snake venom serine protease | 93 | 1729 | 86.7 | 4.65 |
|  | sp\|E5L0E5.1\|VSPPA_AGKPL | Venom plasminogen activator | 76 | 28060 | 4.3 | 5.78 |
|  | sp\|Q8AY78.1\|VSP5M_TRIST | Snake venom serine protease 5 | 67 | 28117 | 4.3 | 7.08 |
|  | sp\|Q5W958.1\|VSP20_BOTJA | Venom serine proteinase-like HS120 | 56 | 27797 | 4.3 | 8.71 |
|  | sp\|Q71QH7.1\|VSPP_TRIST | Snake venom serine protease PA | 54 | 27933 | 4.3 | 5.66 |
|  | sp\|Q8AY79.1\|VSPS2_TRIST | Beta-fibrinogenase stejnefibrase-2 (Snake venom serine protease) | 52 | 28010 | 4.3 | 5.37 |
| 36 | JAS05372.1 | Serine proteinase 9c [*Sistrurus miliarius barbouri*] | 141 | 28221 | 15.4 | 7.53 |
|  | JAS05371.1 | Serine proteinase 9d [*Sistrurus miliarius barbouri*] | 138 | 28266 | 23.6 | 7.04 |
|  | JAV51414.1 | Serine proteinase 8 [*Agkistrodon contortrix contortrix*] | 102 | 28242 | 12.5 | 8.42 |
|  | sp\|P0DMH6.1\|VSP_BOTFO | Snake venom serine protease | 88 | 1729 | 86.7 | 4.65 |
|  | sp\|P0C5B4.2\|VSPGL_GLOSH | Thrombin-like enzyme gloshedobin (Fibrinogen-clotting enzyme/Snake venom serine protease) | 72 | 28597 | 7.7 | 8.42 |
|  | ADI47574.1 | Serine protease, partial *[Echis coloratus*] | 69 | 28437 | 9.2 | 8.7 |
|  | sp\|Q8AY78.1\|VSP5M_TRIST | Snake venom serine protease 5 | 68 | 28117 | 4.3 | 7.08 |
|  | sp\|Q5W958.1\|VSP20_BOTJA | Venom serine proteinase-like HS120 | 62 | 27797 | 4.3 | 8.71 |
|  | sp\|Q8AY79.1\|VSPS2_TRIST | Beta-fibrinogenase stejnefibrase-2 (Snake venom serine protease) | 55 | 28010 | 4.3 | 5.37 |
|  | sp\|Q8UUJ2.2\|VSPUI_GLOUS | Snake venom serine protease ussurin; | 55 | 26184 | 6.8 | 8 |
|  | sp\|Q71QH7.1\|VSPP_TRIST | Snake venom serine protease PA | 52 | 27933 | 4.3 | 5.66 |
|  | XP_032092228.1 | Vitelline membrane outer layer protein 1 homolog isoform X1 [*Thamnophis elegans*] | 44 | 21236 | 18.9 | 8.44 |
|  | JAI10638.1 | Vacuolar protein sorting-associated protein 18 homolog [*Crotalus adamanteus*] | 43 | 111967 | 3.4 | 5.55 |
| 37 | JAS05372.1 | Serine proteinase 9c [*Sistrurus miliarius barbouri*] | 150 | 28221 | 15.4 | 7.53 |
|  | JAS05371.1 | Serine proteinase 9d [*Sistrurus miliarius barbouri*] | 124 | 28266 | 23.6 | 7.04 |
|  | sp\|P0DMH6.1\|VSP_BOTFO | Snake venom serine protease | 99 | 1729 | 86.7 | 4.65 |
|  | JAV51414.1 | Serine proteinase 8 *[Agkistrodon contortrix contortrix*] | 96 | 28242 | 12.5 | 8.42 |
|  | sp\|Q9PT41.1\|VSPF5_MACLB | Factor V activator (Lebetina viper venom FV activator/Snake venom serine protease | 69 | 28577 | 7.3 | 8.81 |
|  | ADI47574.1 | Serine protease, partial [*Echis coloratus*] | 68 | 28437 | 9.2 | 8.7 |
|  | sp\|Q8AY78.1\|VSP5M_TRIST | Snake venom serine protease 5; | 67 | 28117 | 4.3 | 7.08 |
|  | XP_023086434.2 | Disintegrin and metalloproteinase domain-containing protein 20-like [*Piliocolobus tephrosceles*] | 58 | 84212 | 5.9 | 6.12 |
|  | sp\|Q8AY79.1\|VSPS2_TRIST | Beta-fibrinogenase stejnefibrase-2 (Snake venom serine protease) | 54 | 28010 | 4.3 | 5.37 |
| 38 | sp\|Q71QH7.1\|VSPP_TRIST | Snake venom serine protease PA | 89 | 27933 | 8.5 | 5.66 |
|  | sp\|P0DMH6.1\|VSP_BOTFO | Snake venom serine protease | 84 | 1729 | 86.7 | 4.65 |
|  | sp\|E5L0E5.1\|VSPPA_AGKPL | Venom plasminogen activator (Snake venom serine protease) | 75 | 28060 | 4.3 | 5.78 |
|  | sp\|Q5W958.1\|VSP20_BOTJA | Venom serine proteinase-like HS120 (Snake venom serine protease homolog) | 56 | 27797 | 4.3 | 8.71 |
|  | sp\|K4LLQ2.1\|VSP_BOTBA | Thrombin-like enzyme barnettobin (Snake venom serine protease) | 54 | 27567 | 4.4 | 6.25 |
| 39 | sp\|Q71QH7.1\|VSPP_TRIST | Snake venom serine protease PA | 86 | 27933 | 8.5 | 5.66 |
|  | sp\|E5L0E5.1\|VSPPA_AGKPL | Venom plasminogen activator (Snake venom serine protease) | 80 | 28060 | 4.3 | 5.78 |
|  | sp\|Q5W958.1\|VSP20_BOTJA | Venom serine proteinase-like HS120 (Snake venom serine protease homolog) | 63 | 27797 | 4.3 | 8.71 |
|  | sp\|K4LLQ2.1\|VSP_BOTBA | Thrombin-like enzyme barnettobin (Snake venom serine protease) | 58 | 27567 | 4.4 | 6.25 |
|  | sp\|P0DMH6.1\|VSP_BOTFO | Snake venom serine protease | 52 | 1729 | 86.7 | 4.65 |
| 40 | sp\|E5L0E5.1\|VSPPA_AGKPL | Venom plasminogen activator (Snake venom serine protease) | 46 | 28060 | 4.3 | 5.78 |
| 41 | - | Not identified |  |  |  |  |
| 42 | JAS05371.1 | Serine proteinase 9d [*Sistrurus miliarius barbouri*] | 85 | 28266 | 12.7 | 7.04 |
|  | sp\|P0DMH6.1\|VSP_BOTFO | Snake venom serine protease | 85 | 1729 | 86.7 | 4.65 |
|  | sp\|Q8AY78.1\|VSP5M_TRIST | Snake venom serine protease 5 | 58 | 28117 | 4.3 | 7.08 |
|  | XP_032085798.1 | 60S ribosomal protein L6 isoform X1 [*Thamnophis elegans*] | 54 | 30312 | 11.3 | 10.89 |
|  | sp\|Q5W958.1\|VSP20_BOTJA | Venom serine proteinase-like HS120 (Snake venom serine protease homolog) | 50 | 27797 | 4.3 | 8.71 |
|  | sp\|Q8AY79.1\|VSPS2_TRIST | Beta-fibrinogenase stejnefibrase-2 (Snake venom serine protease) | 46 | 28010 | 4.3 | 5.37 |
|  | sp\|Q71QH7.1\|VSPP_TRIST | Snake venom serine protease PA | 43 | 27933 | 4.3 | 5.66 |
| 43 | pdb\|1BQY\|A | Chain A, Plasminogen Activator | 205 | 25590 | 16.7 | 5.71 |
|  | sp\|E5L0E5.1\|VSPPA_AGKPL | Venom plasminogen activator (Snake venom serine protease) | 163 | 28060 | 8.1 | 5.78 |
|  | JAS04407.1 | Serine proteinase 6 [*Agkistrodon piscivorus conanti*] | 138 | 28115 | 7.4 | 5.54 |
|  | JAS04415.1 | Serine proteinase 19b [*Agkistrodon piscivorus conanti*] | 84 | 27782 | 7.4 | 5.62 |
|  | sp\|Q072L7.1\|VSP_LACST | Snake venom serine protease | 77 | 27796 | 9.7 | 6.97 |
|  | sp\|O13069.1\|VSP2_BOTJA | Thrombin-like enzyme KN-BJ 2 (Kinin-releasing and fibrinogen-clotting serine protease 2) | 72 | 27876 | 4.3 | 8.68 |
| 44 | pdb\|1BQY\|A | Chain A, Plasminogen Activator | 248 | 25590 | 19.2 | 5.71 |
|  | JAS04429.1 | Serine proteinase 13e [*Agkistrodon piscivorus conanti]* | 194 | 27985 | 9.3 | 5.95 |
|  | sp\|E5L0E5.1\|VSPPA_AGKPL | Venom plasminogen activator (Snake venom serine protease) | 190 | 28060 | 8.1 | 5.78 |
|  | JAS04407.1 | Serine proteinase 6 [*Agkistrodon piscivorus conanti*] | 144 | 28115 | 7.4 | 5.54 |
|  | JAS04415.1 | Serine proteinase 19b [*Agkistrodon piscivorus conanti*] | 100 | 27782 | 7.4 | 5.62 |
|  | sp\|Q27J47.1\|VSPPA_LACMU | Venom plasminogen activator LV-PA | 93 | 28044 | 8.5 | 5.97 |
|  | sp\|O13069.1\|VSP2_BOTJA | Thrombin-like enzyme KN-BJ 2 (Kinin-releasing and fibrinogen-clotting serine protease 2) | 63 | 27876 | 4.3 | 8.68 |
|  | XP_026523831.1 | Integrin alpha-4 [*Notechis scutatus*] | 54 | 114850 | 1.2 | 6.22 |
| 45 | JAS04417.1 | Serine proteinase 18b [*Agkistrodon piscivorus conanti*] | 158 | 27728 | 11.6 | 5.67 |
|  | pdb\|1BQY\|A | Chain A, Plasminogen Activator | 146 | 25590 | 13.2 | 5.71 |
|  | JAS04757.1 | Serine proteinase 1f [*Crotalus horridus*] | 134 | 28133 | 12 | 5.73 |
|  | JAS04415.1 | Serine proteinase 19b [*Agkistrodon piscivorus conanti*] | 124 | 27782 | 11.6 | 5.62 |
|  | sp\|O13069.1\|VSP2_BOTJA | Thrombin-like enzyme KN-BJ 2 (Kinin-releasing and fibrinogen-clotting serine protease 2) | 113 | 27876 | 7 | 8.68 |
|  | sp\|Q6T5L0.2\|VSPSH_GLOSH | Alpha-fibrinogenase shedaoenase (Snake venom serine protease) | 112 | 26399 | 13 | 6.7 |
|  | JAS04429.1 | Serine proteinase 13e [*Agkistrodon piscivorus conanti*] | 103 | 27985 | 8.9 | 5.95 |
|  | sp\|A8QL56.1\|VSP1_OPHHA | Alpha- and beta-fibrinogenase OhS1 | 64 | 28637 | 4.2 | 5.4 |
|  | AAZ75628.1 | Kallikrein-Phi4, partial [*Philodryas olfersi*i] | 63 | 26827 | 4.9 | 5.88 |
|  | sp\|E5AJX2.1\|VSP_VIPBN | Snake venom serine protease nikobin | 53 | 28197 | 8.9 | 7.96 |
|  | QHR82809.1 | Serine protease 2 [*Vipera anatolica senliki*] | 53 | 28084 | 8.9 | 8.77 |
|  | JAI12774.1 | Leucine-rich repeat-containing protein 7-like [*Crotalus adamanteus*] | 47 | 163679 | 1 | 6.72 |
|  | sp\|C0HLA2.1\|VSP3_LACMR | Thrombin-like enzyme LmrSP-3Sn (ake venom serine protease) | 44 | 2942 | 50 | 4.1 |
|  | JAS04670.1 | Serine proteinase 3c [*Crotalus adamanteus*] | 42 | 28849 | 6.9 | 5.35 |
| 46 | pdb\|1BQY\|A | Chain A, Plasminogen Activator | 272 | 25590 | 19.2 | 5.71 |
|  | JAS04757.1 | Serine proteinase 1f [*Crotalus horridus*] | 250 | 28133 | 12.8 | 5.73 |
|  | JAS04429.1 | Serine proteinase 13e [*Agkistrodon piscivorus conanti*] | 213 | 27985 | 9.7 | 5.95 |
|  | JAS04417.1 | Serine proteinase 18b [*Agkistrodon piscivorus conanti*] | 195 | 27728 | 12 | 5.67 |
|  | JAS04415.1 | Serine proteinase 19b [*Agkistrodon piscivorus conant*i] | 164 | 27782 | 12 | 5.62 |
|  | sp\|Q072L7.1\|VSP_LACST | Snake venom serine protease | 148 | 27796 | 9.3 | 6.97 |
|  | sp\|O13069.1\|VSP2_BOTJA | Thrombin-like enzyme KN-BJ 2 (Kinin-releasing and fibrinogen-clotting serine protease 2) | 108 | 26399 | 13 | 6.7 |
|  | pdb\|4E7N\|A | Chain A, Snake-venom Thrombin-like Enzyme | 98 | 28333 | 8.5 | 8.43 |
|  | XP_032089049.1 | Spectrin alpha chain, non-erythrocytic 1 [*Thamnophis elegans*] | 54 | 263010 | 1.3 | 5.28 |
|  | XP_032064352.1 | Zinc finger protein 347-like [*Thamnophis elegans*] | 49 | 169270 | 1.8 | 9 |
|  | sp\|Q9PT41.1\|VSPF5_MACLB | Factor V activator/Lebetina viper venom FV activatorSnake venom serine protease | 45 | 28577 | 5.4 | 8.81 |
|  | ADI47574.1 | Serine protease, partial [*Echis coloratus*] | 44 | 28437 | 7.3 | 8.7 |
| 47 | JAS04757.1 | Serine proteinase 1f [*Crotalus horridus*] | 287 | 28133 | 12.8 | 5.73 |
|  | pdb\|1BQY\|A | Chain A, Plasminogen Activator | 277 | 25590 | 19.2 | 5.71 |
|  | JAS04417.1 | Serine proteinase 18b [*Agkistrodon piscivorus conanti*] | 235 | 27728 | 12 | 5.67 |
|  | JAS04429.1 | Serine proteinase 13e [*Agkistrodon piscivorus conant*i] | 229 | 27985 | 9.7 | 5.95 |
|  | JAS04415.1 | Serine proteinase 19b [*Agkistrodon piscivorus conanti*] | 196 | 27782 | 12 | 5.62 |
|  | JAV01826.1 | BATXSVSP10 [Bothrops atrox] | 174 | 28606 | 15 | 8.81 |
|  | pdb\|4E7N\|A | Chain A, Snake-venom Thrombin-like Enzyme | 146 | 26370 | 13 | 6.41 |
|  | sp\|Q6T5L0.2\|VSPSH_GLOSH | Alpha-fibrinogenase shedaoenase (Snake venom serine protease) | 145 | 26399 | 13 | 6.7 |
|  | sp\|O13069.1\|VSP2_BOTJA | Thrombin-like enzyme KN-BJ 2 (Kinin-releasing and fibrinogen-clotting serine protease 2) | 122 | 27876 | 7 | 8.68 |
|  | sp\|Q71QI0.1\|VSP07_TRIST | Snake venom serine protease homolog KN7 | 117 | 28703 | 20 | 9.26 |
|  | XP_015671556.1 | Snake venom serine protease [*Protobothrops mucrosquamatus*] | 106 | 28023 | 9.3 | 5.69 |
|  | JAS04671.1 | Serine proteinase 3b [*Crotalus adamanteus*] | 86 | 28890 | 11.5 | 5.51 |
|  | QHR82809.1 | Serine protease 2 [*Vipera anatolica senliki*] | 81 | 28084 | 5.8 | 8.77 |
|  | sp\|A8QL53.1\|VSP1_NAJAT | Snake venom serine protease NaSP | 55 | 31117 | 3.2 | 6.49 |
|  | XP_026523831.1 | Integrin alpha-4 [*Notechis scutatus*] | 42 | 114850 | 1.2 | 6.22 |
| 48 | pdb\|1BQY\|A | Chain A, Plasminogen Activator | 194 | 25590 | 16.7 | 5.71 |
|  | JAS04407.1 | Serine proteinase 6 [*Agkistrodon piscivorus conanti*] | 167 | 28115 | 7.4 | 5.54 |
|  | sp\|E5L0E5.1\|VSPPA_AGKPL | Venom plasminogen activator (Snake venom serine protease) | 163 | 28060 | 8.1 | 5.78 |
|  | JAS04415.1 | Serine proteinase 19b [*Agkistrodon piscivorus conanti*] | 132 | 27782 | 7.4 | 5.62 |
|  | sp\|Q072L7.1\|VSP_LACST | Snake venom serine protease | 92 | 27796 | 9.7 | 6.97 |
|  | XP_026523831.1 | Integrin alpha-4 [*Notechis scutatus*] | 54 | 114850 | 1.2 | 6.22 |
|  | ETE59238.1 | Fascin-3, partial [*Ophiophagus hannah*] | 51 | 14941 | 27.8 | 9.06 |
| 49 | JAV01826.1 | BATXSVSP10 [*Bothrops atrox*] | 268 | 28606 | 15.8 | 8.81 |
|  | sp\|P81176.1\|VSP1_GLOBL | Thrombin-like enzyme halystase (Snake venom serine protease) | 262 | 26466 | 23.1 | 6.71 |
|  | JAS04411.1 | Serine proteinase 2 [*Agkistrodon piscivorus conanti*] | 259 | 28333 | 15.5 | 8.43 |
|  | sp\|O93421.2\|VSPPE_GLOHA | Snake venom serine protease pallase | 258 | 26031 | 25.8 | 5.9 |
|  | sp\|Q6T5L0.2\|VSPSH_GLOSH | Alpha-fibrinogenase shedaoenase (Snake venom serine protease) | 256 | 26399 | 23.1 | 6.7 |
|  | JAS04417.1 | Serine proteinase 18b [*Agkistrodon piscivorus conanti*] | 209 | 27728 | 11.6 | 5.67 |
|  | JAS04670.1 | Serine proteinase 3c [*Crotalus adamanteus*] | 202 | 28849 | 9.2 | 5.35 |
|  | sp\|Q072L7.1\|VSP_LACST | Snake venom serine protease | 198 | 27796 | 8.9 | 6.97 |
|  | sp\|Q9YGJ2.1\|VSP1_GLOHA | Snake venom serine protease pallabin | 184 | 28662 | 16.2 | 5.8 |
|  | JAS04415.1 | Serine proteinase 19b [*Agkistrodon piscivorus conanti*] | 173 | 27782 | 11.6 | 5.62 |
|  | sp\|O13069.1\|VSP2_BOTJA | Thrombin-like enzyme KN-BJ 2 (Kinin-releasing and fibrinogen-clotting serine protease 2) | 173 | 27876 | 7.8 | 8.68 |
|  | sp\|Q7SZE2.1\|VSPD_GLOUS | Bradykinin-releasing enzyme KR-E-1 (Snake venom serine protease) | 165 | 25335 | 19.7 | 4.82 |
|  | sp\|Q91053.1\|VSP1_GLOUS | Thrombin-like enzyme calobin-1 (Snake venom serine protease) | 163 | 28889 | 16 | 6.38 |
|  | JAS04757.1 | Serine proteinase 1f [*Crotalus horridus*] | 144 | 28133 | 12 | 5.73 |
|  | sp\|A8QL56.1\|VSP1_OPHHA | Alpha- and beta-fibrinogenase OhS1 | 108 | 28637 | 5.8 | 5.4 |
|  | sp\|Q71QJ4.1\|VSP04_TRIST | Snake venom serine protease homolog KN4 | 107 | 28685 | 9.2 | 8.45 |
|  | AAZ75628.1 | Kallikrein-Phi4, partial [*Philodryas olfersii*] | 106 | 26827 | 6.6 | 5.88 |
|  | pdb\|1BQY\|A | Chain A, Plasminogen Activator | 102 | 28029 | 7.8 | 7.51 |
|  | sp\|A0A1I9KNP0.1\|VSPH1_VIPAA | Vaa serine proteinase homolog 1 | 96 | 28909 | 10 | 8.79 |
|  | JAS04742.1 | Serine proteinase 9d [*Crotalus horridus*] | 92 | 28299 | 12.7 | 6.64 |
|  | sp\|J3S833.1\|VSP2_CROAD | Snake venom serine proteinase 2 | 78 | 28298 | 15.1 | 8.69 |
|  | JAS05249.1 | Serine proteinase 2 [*Sistrurus tergeminus*] | 76 | 28326 | 7.3 | 8.23 |
|  | sp\|C0HLA2.1\|VSP3_LACMR | Thrombin-like enzyme LmrSP-3 (Snake venom serine protease) | 76 | 2942 | 50 | 4.1 |
|  | sp\|E5AJX2.1\|VSP_VIPBN | Snake venom serine protease nikobin; S | 72 | 28197 | 3.5 |  |
|  | XP_039181676.1 | Snake venom serine protease-like isoform X1 [*Crotalus tigris*] | 64 | 25811 | 8.1 | 9.61 |
|  | pdb\|1OP0\|A | Chain A, Venom serine proteinase | 63 | 25318 | 5.6 | 5.29 |
|  | sp\|A0A024BTN9.1\|OXLA_BOTSC | L-amino acid oxidase Bs29 | 43 | 56341 | 2.4 | 5.79 |
| 50 | JAV01826.1 | BATXSVSP10 [Bothrops atrox] | 208 | 28606 | 15.8 | 8.81 |
|  | pdb\|4E7N\|A | Chain A, Snake-venom Thrombin-like Enzyme | 182 | 26370 | 13.9 | 6.41 |
|  | JAS04411.1 | Serine proteinase 2 [*Agkistrodon piscivorus conanti*] | 180 | 28333 | 9.3 | 8.43 |
|  | sp\|Q6T5L0.2\|VSPSH_GLOSH | Alpha-fibrinogenase shedaoenase | 165 | 26399 | 13.9 | 6.7 |
|  | JAS04670.1 | Serine proteinase 3c [*Crotalus adamanteus*] | 164 | 28849 | 9.2 | 5.35 |
|  | sp\|Q072L7.1\|VSP_LACST | Snake venom serine protease | 144 | 27796 | 10.1 | 6.97 |
|  | sp\|O13069.1\|VSP2_BOTJA | Thrombin-like enzyme KN-BJ 2 (Kinin-releasing and fibrinogen-clotting serine protease 2) | 129 | 27876 | 7.8 | 8.68 |
|  | JAS04417.1 | Serine proteinase 18b [*Agkistrodon piscivorus conanti*] | 121 | 27728 | 8.5 | 5.67 |
|  | JAS04415.1 | Serine proteinase 19b [*Agkistrodon piscivorus conanti*] | 116 | 27782 | 8.5 | 5.62 |
|  | sp\|Q71QJ4.1\|VSP04_TRIST | Snake venom serine protease homolog KN4 | 79 | 28685 | 9.2 | 8.45 |
|  | sp\|A8QL56.1\|VSP1_OPHHA | Alpha- and beta-fibrinogenase OhS1 | 54 | 28637 | 4.2 | 5.4 |
|  | AAZ75628.1 | Kallikrein-Phi4, partial [*Philodryas olfersii*] | 54 | 28637 | 4.2 | 5.4 |
|  | XP_026541175.1 | N-acetylated-alpha-linked acidic dipeptidase-like protein [*Notechis scutatus*] | 45 | 81911 | 6.6 | 5.49 |
| 51 | ETE60526.1 | Trichohyalin, partial [*Ophiophagus hannah*] | 45 | 80507 | 4.4 | 6.64 |
| 52 | JAS05359.1 | Cysteine-rich secretory protein 1c [*Sistrurus tergeminus*] | 99 | 26787 | 14.2 | 7.83 |
| 53 | ETE67131.1 | Keratin, type II cytoskeletal 1, partial [*Ophiophagus hannah*] | 179 | 240496 | 1.4 | 6.24 |
| 54 | sp\|P82981.1\|VSP2_AGKCO | Thrombin-like enzyme contortrixobin/Fibrinogen-clotting enzyme (Snake venom serine protease) | 146 | 25396 | 10.3 | 4.95 |
|  | sp\|Q7T229.1\|VSPH_BOTJR | Snake venom serine protease homolog | 116 | 28636 | 11.2 | 8.81 |
|  | JAV51425.1 | Serine proteinase 15a [*Agkistrodon contortrix contortrix*] | 115 | 28940 | 14.9 | 6.3 |
|  | JAS04670.1 | Serine proteinase 3c [*Crotalus adamanteus*] | 78 | 28849 | 6.9 | 5.35 |
|  | JAS04748.1 | Serine proteinase 6 [*Crotalus horridus*] | 62 | 28594 | 10.8 | 8.68 |
|  | XP_025414344.1 | Disintegrin and metalloproteinase domain-containing protein 9 [*Sipha flava*] | 44 | 138126 | 3 | 7.88 |
| 55 | sp\|Q7T229.1\|VSPH_BOTJR | Snake venom serine protease homolog | 150 | 28636 | 11.2 | 8.81 |
|  | JAS04411.1 | Serine proteinase 2 [*Agkistrodon piscivorus conanti*] | 133 | 28333 | 8.5 | 8.43 |
|  | JAS04670.1 | Serine proteinase 3c [*Crotalus adamanteus*] | 117 | 28849 | 8.5 | 5.35 |
|  | sp\|P82981.1\|VSP2_AGKCO | Thrombin-like enzyme contortrixobin/Fibrinogen-clotting enzyme (Snake venom serine protease) | 116 | 25396 | 10.3 | 4.95 |
|  | JAV51425.1 | Serine proteinase 15a [*Agkistrodon contortrix contortrix*] | 105 | 28940 | 14.9 | 6.3 |
|  | BAN82034.1 | Serine protease, partial [*Protobothrops flavoviridis*] | 62 | 22377 | 11 | 8.72 |
|  | JAS04748.1 | Serine proteinase 6 [*Crotalus horridus*] | 55 | 28594 | 10.8 | 8.68 |
|  | sp\|Q71QJ4.1\|VSP04_TRIST | Snake venom serine protease homolog KN4 | 53 | 28685 | 8.5 | 8.45 |
|  | sp\|P85109.1\|VSP1_GLOBR | Thrombin-like enzyme kangshuanmei/Fibrinogen-clotting enzyme (Snake venom serine protease) | 45 | 26415 | 4.7 | 8.27 |
| 56 | sp\|O13057.1\|VSP2_PROFL | Snake venom serine protease 2 | 212 | 28623 | 11.9 | 9.21 |
|  | sp\|Q7T229.1\|VSPH_BOTJR | Snake venom serine protease homolog | 202 | 28636 | 11.9 | 8.81 |
|  | JAV51425.1 | Serine proteinase 15a [*Agkistrodon contortrix contortrix*] | 196 | 28940 | 15.6 | 6.3 |
|  | XP_039181680.1 | Snake venom serine proteinase 12-like [*Crotalus tigris*] | 188 | 24539 | 16.7 | 5.4 |
|  | JAS04670.1 | Serine proteinase 3c [*Crotalus adamanteus*] | 160 | 28849 | 9.2 | 5.35 |
|  | sp\|P82981.1\|VSP2_AGKCO | Thrombin-like enzyme contortrixobin/Fibrinogen-clotting enzyme (Snake venom serine protease) | 143 | 25396 | 10.3 | 4.95 |
|  | sp\|Q6T5L0.2\|VSPSH_GLOSH | Alpha-fibrinogenase shedaoenase | 136 | 26399 | 13.9 | 6.7 |
|  | JAS04748.1 | Serine proteinase 6 [*Crotalus horridus*] | 133 | 28594 | 11.5 | 8.68 |
|  | sp\|Q072L7.1\|VSP_LACST | Snake venom serine protease | 124 | 27796 | 12.4 | 6.97 |
|  | sp\|Q71QJ4.1\|VSP04_TRIST | Snake venom serine protease homolog KN4 | 92 | 28685 | 9.2 | 8.45 |
|  | XP_015671564.1 | Snake venom serine protease serpentokallikrein-1 [*Protobothrops mucrosquamatus*] | 85 | 88822 | 2.2 | 6.65 |
|  | JAV51414.1 | Serine proteinase 8 [Agkistrodon contortrix contortrix] | 72 | 28242 | 7.8 | 8.42 |
|  | sp\|Q5W958.1\|VSP20_BOTJA | Venom serine proteinase-like HS120 (Snake venom serine protease homolog) | 51 | 27797 | 4.3 | 8.71 |
|  | sp\|P85109.1\|VSP1_GLOBR | Thrombin-like enzyme kangshuanmei/Fibrinogen-clotting enzyme (Snake venom serine protease) | 45 | 26415 | 4.7 | 8.27 |
| 57 | sp\|Q7T229.1\|VSPH_BOTJR | Snake venom serine protease homolog | 192 | 28636 | 11.9 | 8.81 |
|  | JAV51425.1 | Serine proteinase 15a [*Agkistrodon contortrix contortrix*] | 169 | 28940 | 15.6 | 6.3 |
|  | JAS04411.1 | Serine proteinase 2 [*Agkistrodon piscivorus conanti*] | 166 | 28333 | 9.3 | 8.43 |
|  | JAS04670.1 | Serine proteinase 3c [Crotalus adamanteus] | 149 | 28849 | 9.2 | 5.35 |
|  | sp\|P82981.1\|VSP2_AGKCO | Thrombin-like enzyme contortrixobin/Fibrinogen-clotting enzyme (Snake venom serine protease) | 137 | 25396 | 10.3 | 4.95 |
|  | JAS04748.1 | Serine proteinase 6 [*Crotalus horridus*] | 116 | 28594 | 11.5 | 8.68 |
|  | sp\|Q71QJ4.1\|VSP04_TRIST | Snake venom serine protease homolog KN4 | 82 | 28685 | 9.2 | 8.45 |
|  | sp\|Q5W958.1\|VSP20_BOTJA | Venom serine proteinase-like HS120 (Snake venom serine protease homolog) | 55 | 27797 | 4.3 | 8.71 |
|  | sp\|Q71QH7.1\|VSPP_TRIST | Snake venom serine protease PA | 46 | 27933 | 4.3 |  |
|  | sp\|P85109.1\|VSP1_GLOBR | Thrombin-like enzyme kangshuanmei/Fibrinogen-clotting enzyme (Snake venom serine protease) | 45 | 26415 | 4.7 | 8.27 |
| 58 | sp\|P82981.1\|VSP2_AGKCO | Thrombin-like enzyme contortrixobin/Fibrinogen-clotting enzyme (Snake venom serine protease) | 113 | 25396 | 10.3 | 4.95 |
|  | sp\|B0ZT25.1\|VSPH_PROJR | Snake venom serine protease homolog | 75 | 28776 | 6.9 | 9.09 |
|  | sp\|J3S832.1\|VSPB_CROAD | Snake venom serine proteinase 11 | 70 | 28033 | 6.2 | 7.01 |
|  | JAS04670.1 | Serine proteinase 3c [*Crotalus adamanteus*] | 58 | 28849 | 6.9 | 5.35 |
|  | sp\|Q072L7.1\|VSP_LACST | Snake venom serine proteaser | 44 | 27796 | 10.9 | 6.97 |
| 59 | JAS05359.1 | Cysteine-rich secretory protein 1c [*Sistrurus tergeminus*] | 353 | 26787 | 15.8 | 7.83 |
|  | BAN82147.1 | Cysteine rich secretory protein [*Ovophis okinavensis*] | 308 | 26920 | 17.1 | 5.97 |
|  | pdb\|1WVR\|A | Chain A, Triflin | 280 | 24782 | 15.8 | 7.03 |
|  | sp\|Q7ZT99.1\|CRVP_CROAT | Cysteine-rich venom protein catrin | 280 | 26629 | 14.6 | 8.42 |
|  | JAS04734.1 | Cysteine-rich secretory protein [*Crotalus adamanteus*] | 244 | 26612 | 11.3 | 7.43 |
|  | sp\|P0DL18.1\|CRVP_OVOOK | Cysteine-rich venom protein okinavin | 64 | 3496 | 33.3 | 5.5 |
|  | XP_032080246.1 | Centromere-associated protein E [*Thamnophis elegans*] | 45 | 308791 | 1.3 | 5.06 |
| 60 | JAS05359.1 | Cysteine-rich secretory protein 1c [*Sistrurus tergeminus*] | 259 | 26787 | 15.8 | 7.83 |
|  | BAN82147.1 | Cysteine rich secretory protein [*Ovophis okinavensis*] | 195 | 26920 | 16.7 | 5.97 |
|  | sp\|Q7ZTA0.1\|CRVP_AGKPI | Cysteine-rich venom protein piscivorin | 188 | 26664 | 19.6 |  |
|  | pdb\|1WVR\|A | Chain A, Triflin | 178 | 24782 | 15.4 | 7.03 |
|  | JAS04734.1 | Cysteine-rich secretory protein [*Crotalus adamanteus*] | 176 | 26612 | 11.3 | 7.43 |
|  | sp\|P0DL18.1\|CRVP_OVOOK | Cysteine-rich venom protein okinavin | 64 | 3496 | 33.3 | 5.5 |
| 61 | JAS05359.1 | Cysteine-rich secretory protein 1c [*Sistrurus tergeminus*] | 399 | 26787 | 23.8 | 7.83 |
|  | JAS05484.1 | Cysteine-rich secretory protein 1b [*Sistrurus miliarius barbouri*] | 399 | 26772 | 23.8 | 7.41 |
|  | BAN82147.1 | Cysteine rich secretory protein [*Ovophis okinavensis*] | 398 | 26920 | 25.4 | 5.97 |
|  | sp\|Q7ZTA0.1\|CRVP_AGKPI | Cysteine-rich venom protein piscivorin | 318 | 26664 | 25.4 | 7.83 |
|  | pdb\|1WVR\|A | Chain A, Triflin | 310 | 24782 | 21.3 | 7.03 |
|  | JAS04734.1 | Cysteine-rich secretory protein [*Crotalus adamanteus*] | 234 | 26612 | 11.3 | 7.43 |
|  | sp\|P0DJG8.1\|CRVP_HELAG | Helicopsin | 91 | 2618 | 61.9 | 4.79 |
|  | sp\|P0DL18.1\|CRVP_OVOOK | Cysteine-rich venom protein okinavin | 64 | 3496 | 33.3 | 5.5 |
| 62 | JAS05359.1 | Cysteine-rich secretory protein 1c [*Sistrurus tergeminus*] | 335 | 26787 | 14.2 | 7.83 |
|  | BAN82147.1 | Cysteine rich secretory protein [*Ovophis okinavensis*] | 293 | 26920 | 23.3 | 5.97 |
|  | pdb\|1WVR\|A | Chain A, Triflin | 257 | 24782 | 19.5 | 7.03 |
|  | sp\|Q7ZTA0.1\|CRVP_AGKPI | Cysteine-rich venom protein piscivorin | 256 | 26664 | 18.3 | 7.83 |
|  | JAS04734.1 | Cysteine-rich secretory protein [*Crotalus adamanteus*] | 239 | 26612 | 11.3 | 7.43 |
| 63 | XP_028906446.1 | Disintegrin and metalloproteinase domain-containing protein 17 [*Ornithorhynchus anatinus*] | 43 | 94942 | 2.6 | 6.24 |
| 64 | XP_023418723.1 | Disintegrin and metalloproteinase domain-containing protein 17 [*Cavia porcellus*] | 52 | 92703 | 3.7 | 5.55 |
| 65 | XP_024069019.3 | Disintegrin and metalloproteinase domain-containing protein 17 [*Terrapene carolina triunguis*] | 49 | 99769 | 2.1 | 5.98 |
| 66 | sp\|P0DJJ7.1\|PA2A_OVOMO | Acidic phospholipase A2 Omo-E6 | 59 | 3261 | 37.9 | 6.02 |
|  | XP_026535629.1 | Dynein heavy chain 8, axonemal [*Notechis scutatus*] | 45 | 511281 | 0.8 | 5.92 |
| 67 | sp\|A8E2V8.1\|PA2A_TRIGS | Acidic phospholipase A2 Tgc-E6 | 171 | 15678 | 18 | 4.73 |
|  | sp\|P0DJJ7.1\|PA2A_OVOMO | Acidic phospholipase A2 Omo-E6 | 112 | 3261 | 51.7 | 6.02 |
|  | JAV51451.1 | Phospholipase A2 1a [*Agkistrodon contortrix contortri*x] | 93 | 15952 | 12.9 | 8.61 |
|  | pdb\|3JR8\|A | Chain A, Phospholipase A2 bothropstoxin-2 | 73 | 13985 | 21.3 | 8.5 |
|  | sp\|Q7ZTA6.1\|PA2AB_CROVV | Acidic phospholipase A2 Cvv-E6b | 68 | 15429 | 13 | 5.39 |
|  | sp\|C0HJC1.1\|PA2_BOTLA | Acidic phospholipase A2 BlatPLA2 | 63 | 13881 | 20.5 | 4.66 |
|  | pdb\|1GMZ\|A | Chain A, Phospholipase A2 | 59 | 13850 | 13.9 | 8.5 |
|  | JAS04568.1 | Phospholipase A2 1b [*Boiga irregularis*] | 55 | 16906 | 4.6 | 8.59 |
| 68 | AAB28455.1 | Phospholipase A2 isozyme III, PLA2-III {EC 3.1.1.4} [*Trimeresurus gramineus*] | 77 | 13716 | 18 | 4.71 |
|  | sp\|Q7ZTA6.1\|PA2AB_CROVV | Acidic phospholipase A2 Cvv-E6b | 63 | 15429 | 13 | 5.39 |
|  | sp\|P0DJJ7.1\|PA2A_OVOMO | Acidic phospholipase A2 Omo-E6 | 55 | 3261 | 37.9 | 6.02 |
| 69 | sp\|P0DJJ7.1\|PA2A_OVOMO | Acidic phospholipase A2 Omo-E6 | 56 | 3261 | 37.9 | 6.02 |
| 70 | - | Not identified |  |  |  |  |
| 71 | - | Not identified |  |  |  |  |
| 72 | pdb\|1BK9\|A | Chain A, Phospholipase A2 | 88 | 13964 | 14.5 | 4.82 |
|  | JAV51451.1 | Phospholipase A2 1a [*Agkistrodon contortrix contortrix*] | 88 | 15952 | 12.9 | 8.61 |
|  | AAB28455.1 | Phospholipase A2 isozyme III, PLA2-III {EC 3.1.1.4} [*Trimeresurus gramineus*] | 74 | 13716 | 18 | 4.71 |
|  | JAS04568.1 | Phospholipase A2 1b [*Boiga irregularis*] | 56 | 16906 | 4.6 | 8.59 |
|  | sp\|P0DJJ7.1\|PA2A_OVOMO | Acidic phospholipase A2 Omo-E6 | 53 | 3261 | 37.9 | 6.02 |
|  | ETE61374.1 | Dynein heavy chain 8, axonemal [*Ophiophagus hannah*] | 52 | 284552 | 1.3 | 5.91 |
| 73 | sp\|A8E2V8.1\|PA2A_TRIGS | Acidic phospholipase A2 Tgc-E6 | 166 | 15678 | 18 | 4.73 |
|  | sp\|P0DJJ7.1\|PA2A_OVOMO | Acidic phospholipase A2 Omo-E6 | 125 | 3261 | 51.7 | 6.02 |
|  | JAV51451.1 | Phospholipase A2 1a [*Agkistrodon contortrix contortrix*] | 101 | 15952 | 12.9 | 8.61 |
|  | sp\|Q7ZTA6.1\|PA2AB_CROVV | Acidic phospholipase A2 Cvv-E6b | 61 | 15429 | 13 | 5.39 |
|  | sp\|C0HJC1.1\|PA2_BOTLA | Acidic phospholipase A2 BlatPLA2 | 59 | 13881 | 20.5 | 4.66 |
|  | JAS04568.1 | Phospholipase A2 1b [*Boiga irregularis*] | 55 | 16906 | 4.6 | 8.59 |
| 74 | sp\|A8E2V8.1\|PA2A_TRIGS | Acidic phospholipase A2 Tgc-E6 | 172 | 15678 | 18 | 4.73 |
|  | sp\|P0DJJ7.1\|PA2A_OVOMO | Acidic phospholipase A2 Omo-E6 | 122 | 3261 | 51.7 | 6.02 |
|  | JAV51451.1 | Phospholipase A2 1a [*Agkistrodon contortrix contortrix*] | 90 | 15952 | 12.9 | 8.61 |
|  | sp\|Q6EAN6.1\|PA2A_SISTE | Acidic phospholipase A2 homolog sistruxin APrecursor | 76 | 15419 | 34.1 | 4.44 |
|  | XP_032088152.1 | Group IIE secretory phospholipase A2-like [*Thamnophis elegans*] | 66 | 17310 | 12.8 | 8.62 |
|  | sp\|Q7ZTA6.1\|PA2AB_CROVV | Acidic phospholipase A2 Cvv-E6b | 63 | 15429 | 13 | 5.39 |
|  | AAB28455.1 | Phospholipase A2 isozyme III, PLA2-III {EC 3.1.1.4} [Trimeresurus gramineus=green habu snakes, venom, Peptide, 122 aa] | 59 | 13716 | 18 | 4.71 |
|  | JAV01879.1 | BATXPLA5 [Bothrops atrox] | 56 | 15504 | 13 | 6.67 |
|  | sp\|P06860.1\|PA2BX_PROFL | RecName: Full=Basic phospholipase A2 PL-X; Short=svPLA2; AltName: Full=Phosphatidylcholine 2-acylhydrolase | 55 | 13971 | 5.7 | 8.5 |
|  | AAB28454.1 | Phospholipase A2 isozyme IV, PLA2-IV {EC 3.1.1.4} [*Trimeresurus gramineus*] | 54 | 13783 | 18 | 4.72 |
|  | sp\|C0HJC1.1\|PA2_BOTLA | Acidic phospholipase A2 BlatPLA2 | 51 | 13881 | 20.5 | 4.66 |
| 75 | sp\|A8E2V8.1\|PA2A_TRIGS | Acidic phospholipase A2 Tgc-E6 | 192 | 15678 | 18 | 4.73 |
|  | sp\|P0DJJ7.1\|PA2A_OVOMO | Acidic phospholipase A2 Omo-E6 | 140 | 3261 | 51.7 | 6.02 |
|  | pdb\|1C1J\|A | Chain A, Basic phospholipase A2 | 138 | 13888 | 26.2 | 8.71 |
|  | JAS04499.1 | Phospholipase A2 1s [*Agkistrodon piscivorus conanti*] | 130 | 15776 | 29.5 | 8.61 |
|  | sp\|P82896.1\|PA2A5_TRIST | Acidic phospholipase A2 5 | 129 | 13870 | 20.5 | 4.72 |
|  | sp\|D6MKR0.1\|PA2A6_CROHD | Acidic phospholipase A2 CH-E6 | 125 | 15498 | 18.1 | 4.72 |
|  | sp\|Q7ZTA6.1\|PA2AB_CROVV | Acidic phospholipase A2 Cvv-E6b | 118 | 15429 | 13 | 5.39 |
|  | JAV51451.1 | Phospholipase A2 1a [*Agkistrodon contortrix contortrix*] | 103 | 15952 | 12.9 | 8.61 |
|  | sp\|P86907.1\|PA2A_BOTAM | Acidic phospholipase A2 | 99 | 13858 | 36.9 | 6.13 |
|  | sp\|C9DPL5.1\|PA2A1_BOTPI | Acidic phospholipase A2 BpirPLA2-I | 85 | 13627 | 22.1 | 4.9 |
|  | sp\|C0HLF0.1\|PA2_POROP | Basic phospholipase A2 | 81 | 14042 | 15.7 | 9.03 |
|  | sp\|C0HJC1.1\|PA2_BOTLA | Acidic phospholipase A2 BlatPLA2 | 80 | 13881 | 20.5 | 4.66 |
|  | sp\|P86456.1\|PA2A4_BOTAL | Acidic phospholipase A2 SpII RP4 | 79 | 13733 | 14.9 | 6.7 |
|  | QHR82796.1 | Phospholipase A2 3 [*Vipera anatolica senliki*] | 56 | 17437 | 9.6 | 8.97 |
| 76 | sp\|P0DJJ7.1\|PA2A_OVOMO | Acidic phospholipase A2 Omo-E6 | 59 | 3261 | 37.9 | 6.02 |
|  | JAS04407.1 | Serine proteinase 6 [*Agkistrodon piscivorus conanti*] | 43 | 28115 | 6.6 | 5.54 |
| 77 | sp\|B0VXW0.1\|OXLA_SISCA | L-amino-acid oxidase | 106 | 58532 | 4.3 | 6.06 |
|  | sp\|P0DJJ7.1\|PA2A_OVOMO | Acidic phospholipase A2 Omo-E6 | 68 | 3261 | 37.9 | 6.02 |
| 78 | sp\|A8E2V8.1\|PA2A_TRIGS | Acidic phospholipase A2 Tgc-E6 | 201 | 15678 | 18 | 4.73 |
|  | sp\|P0DJJ7.1\|PA2A_OVOMO | Acidic phospholipase A2 Omo-E6 | 138 | 3261 | 51.7 | 6.02 |
|  | JAV51451.1 | Phospholipase A2 1a [*Agkistrodon contortrix contortrix*] | 98 | 15952 | 12.9 | 8.61 |
|  | AAB28454.1 | Phospholipase A2 isozyme IV, PLA2-IV {EC 3.1.1.4} [*Trimeresurus gramineus*] | 93 | 13783 | 18 | 4.72 |
|  | sp\|Q7ZTA6.1\|PA2AB_CROVV | Acidic phospholipase A2 Cvv-E6b | 90 | 15429 | 13 | 5.39 |
|  | sp\|C0HJC1.1\|PA2_BOTLA | Acidic phospholipase A2 BlatPLA2 | 74 | 13881 | 20.5 | 4.66 |
|  | sp\|P86907.1\|PA2A_BOTAM | Acidic phospholipase A2 | 64 | 13858 | 23.8 | 6.13 |
|  | JAS04568.1 | Phospholipase A2 1b [*Boiga irregularis*] | 55 | 16906 | 4.6 | 8.59 |
| 79 | sp\|P0DJJ7.1\|PA2A_OVOMO | Acidic phospholipase A2 Omo-E6 | 80 | 3261 | 51.7 | 6.02 |
|  | sp\|Q7ZTA6.1\|PA2AB_CROVV | Acidic phospholipase A2 Cvv-E6b | 67 | 15429 | 13 | 5.39 |
|  | sp\|P86907.1\|PA2A_BOTAM | Acidic phospholipase A2 | 45 | 13858 | 23.8 | 6.13 |
| 80 | BAN82001.1 | Galactose binding lectin, partial [*Protobothrops flavoviridis*] | 279 | 17654 | 25 | 5.72 |
|  | sp\|P0DM36.1\|LECG_AGKPI | C-type lectin APL | 239 | 16195 | 33.3 | 5.17 |
|  | JAV51455.1 | C-type lectin 9a [*Agkistrodon contortrix contortrix*] | 239 | 18657 | 29.1 | 6.19 |
|  | pdb\|1JZN\|A | Chain A, Galactose-specific lectin | 171 | 16281 | 21.5 | 5.54 |
|  | BAN82148.1 | Galactose binding lectin [*Ovophis okinavensis*] | 169 | 18480 | 13.4 | 5.36 |
|  | XP_026527653.1 | Laminin subunit alpha-1 [*Notechis scutatu*s] | 46 | 331363 | 1 | 6.13 |
|  | XP_026540213.1 | Regulatory solute carrier protein family 1 member 1 [*Notechis scutatus*] | 43 | 37705 | 5.8 | 4.47 |
| 81 | sp\|P81114.1\|SLA4_TRIAB | Snaclec alboaggregin-A subunit beta | 84 | 14357 | 9.8 | 4.62 |
|  | XP_026523846.1 | Titin isoform X1 [*Notechis scutatus*] | 54 | 3675875 | 0.3 | 6.29 |
|  | XP_032085798.1 | 60S ribosomal protein L6 isoform X1 [*Thamnophis elegans*] | 51 | 30312 | 7.5 | 10.89 |
|  | XP_032088226.1 | Forkhead-associated domain-containing protein 1 [*Thamnophis elegans*] | 45 | 137802 | 2.8 | 7.4 |
| 82 | BAN82001.1 | Galactose binding lectin, partial [*Protobothrops flavoviridis*] | 291 | 17654 | 25 | 5.72 |
|  | sp\|P0DM36.1\|LECG_AGKPI | C-type lectin APL | 234 | 16195 | 33.3 | 5.17 |
|  | JAV51455.1 | C-type lectin 9a [*Agkistrodon contortrix contortrix*] | 234 | 18657 | 29.1 | 6.19 |
|  | pdb\|1JZN\|A | Chain A, Galactose-specific lectin | 186 | 16281 | 21.5 | 5.54 |
|  | BAN82148.1 | Galactose binding lectin [*Ovophis okinavensis*] | 170 | 18480 | 13.4 | 5.36 |
| 83 | sp\|P81114.1\|SLA4_TRIAB | Snaclec alboaggregin-A subunit beta | 51 | 14357 | 7.3 | 4.62 |
| 84 | BAN82001.1 | Galactose binding lectin, partial [*Protobothrops flavoviridis*] | 186 | 17654 | 24.3 | 5.72 |
|  | sp\|P0DM36.1\|LECG_AGKPI | C-type lectin APL | 153 | 16195 | 32.6 | 5.17 |
|  | XP_023086434.2 | Disintegrin and metalloproteinase domain-containing protein 20-like [*Piliocolobus tephrosceles*] | 51 | 84212 | 5.1 | 6.12 |
|  | XP_032080033.1 | Biorientation of chromosomes in cell division protein 1-like 1 isoform X1 [*Thamnophis elegans*] | 45 | 319820 | 1.2 | 5.26 |
| 85 | BAN82001.1 | Galactose binding lectin, partial [*Protobothrops flavoviridis*] | 250 | 17654 | 25 | 5.72 |
|  | sp\|P0DM36.1\|LECG_AGKPI | C-type lectin APL | 197 | 16195 | 33.3 | 5.17 |
|  | JAV51455.1 | C-type lectin 9a [Agkistrodon contortrix contortrix] | 197 | 18657 | 29.1 | 6.19 |
|  | XP_032078796.1 | Laminin subunit alpha-1 [*Thamnophis elegans*] | 48 | 339446 | 0.9 | 6.37 |
|  | XP_026540424.1 | Inositol hexakisphosphate and diphosphoinositol-pentakisphosphate kinase 1 isoform X1 [Notechis scutatus] | 44 | 135476 | 1.7 | 5.51 |
| 86 | sp\|P81114.1\|SLA4_TRIAB | Snaclec alboaggregin-A subunit beta | 51 | 14357 | 7.3 | 4.62 |
|  | ETE64295.1 | Glycerol-3-phosphate acyltransferase 4 [*Ophiophagus hannah*] | 47 | 50772 | 8.2 | 8.98 |
| 87 | BAN82149.1 | C-type lectin alpha subunit [*Ovophis okinavensis*] | 106 | 17686 | 14 | 7.51 |
|  | JAS05472.1 | C-type lectin 2 [*Sistrurus miliarius barbouri*] | 50 | 18147 | 16.5 | 7.51 |
|  | ETE66458.1 | Helicase SRCAP, partial [*Ophiophagus hannah*] | 45 | 494261 | 0.7 | 5.25 |
| 88 | BAN82149.1 | C-type lectin alpha subunit [*Ovophis okinavensis*] | 148 | 17686 | 15.9 | 7.51 |
|  | XP_032091805.1 | Glial fibrillary acidic protein [*Thamnophis elegans*] | 62 | 52228 | 5.4 | 5.3 |
| 89 | ETE70787.1 | N6-adenosine-methyltransferase 70 kDa subunit, partial [*Ophiophagus hannah*] | 52 | 59797 | 3.8 | 8.15 |
