# Supplementary material for: Proteomics and immunocharacterization of Asian mountain pit viper (Ovophis monticola) venom
Source: PLoS One. 2021 Dec 1;16(12):e0260496. doi: 10.1371/journal.pone.0260496 (PMC8635378; doi:10.1371/journal.pone.0260496)
Supplement: Attachment — Submitted filename: Response to reviewers.docx [file pone.0260496.s004.docx]

**Responses to reviewers**

**Reviewer #1:**

In this work, the authors demonstrate by venomics approaches, a comprehensive characterization of the venom proteome and immunocharacterization of Asian Mountain pit viper (*Ovophis monticola*) venoms, a medically important snake from Thailand, found at high altitude areas of several mountain ranges of East, South and Southeast Asia. Moreover the authors analyzed the immunoreactivity of monospecific (monovalent) and polyspecific (polyvalent) antivenoms against green pit viper *Trimeresurus albolabris* and hemotoxic venoms, respectively. The authors present a study with satisfactory quality in methodology and data generation. However the manuscript has an inventory of toxins and immunoreactive proteins without coherence with the scientific literature and poor consistency with some cited references. Furthermore, in many topics of the manuscript the authors present a lot of information without any reference. For example, the authors don't compare the results regarding 2D immunoblotting with any previously study. I suggest that this manuscript should be reword to improve the quality about the data presentation.

**Response:** We thank the reviewer for suggestion. We rewrote introduction and discussion to improve data presentation. Relevant references were cited in every part to enhance literature coherence. Biological and geographical details regarding all snakes were also provided in materials and methods (Table 1; page 5, line 105).

- Abstract
Line 33  ..... venoms was investigated by indirect ELISA..... Line 37.....were subjected to 2D immunoblotting
These related methodologies would be more appropriate if they were introduced together.

**Response:** We edited an abstract according to reviewer’s suggestion.

- Introduction
. Line 46-50 - The geographical distribution data deserve references. The same for the sentences introducing biological data about pit viper from lines 51-58.... length of 110 cm.
Lines 59: Incidence of pit vipers  bites, including those from mountain pit vipers, have been documented in their known ranges???.
The lack of references should be revised in all manuscript...
**Response :** We rewrote introduction with relevant references cited in a revised version of manuscript; ref no.6 and 8).

-Materials and methods
Line 92 : Snake venom,
- Are there more information about the venom's sample used in this study? Number of specimens? Localities? size/age...

**Response:** Biological and geographical information regarding all snakes is provided as a table in materials and methods (Table 1; page 5 line 105).

Line 121: ...a non-linear immobilized pH gradient (IPG) strip (pH 3-10 Amersham Bioscience, USA)....
- Why the authors choose the non-linear gradient instead of linear gradient, since the propose were excised immunoreactive spots for mass spectrometric analysis? Just for curious

**Response:** According to our previous experiments, we found that the non-linear immobilized pH gradient (IPG) could provide the well separation of protein in venoms. Therefore, we chose the NL strip for this research.

- Lack references in almost all methods. The references for 2D immunoblotting is required.
**Response:** In a revised materials and methods of manuscripts, we provided relevant references for all methods used. For 2DE immunoblotting, a reference is doi: 10.3390/toxins6051526 as reference page 7, line 137 (ref no.14)

Results
Line 208: Change Ovophis / Ovophis sp or genera Ovophis
- Are there any published information about neutralize activity of monovalent and polyvalent antivenoms against *O. monticola* venom? This information should be provided or I suggest that an experiment to determine the lethal dose 50% should be done.
Table 2. List of identified proteins ... immunologically reacted ...
- If the MW of the spots content reacted/ non-reacted proteins could be provided, this information can improve the quality table.

**Response:** We added a column of MW of proteins in a revised Table 2. In this particular study, we focused mainly on providing the inventory of all protein constituents of Asian mountain pit viper *O. monticola* venom. Those with antigenic properties reacted with monovalent and hematotoxic polyvalent antivenom were given. However, we did not perform neutralizing experiments.

Discussion
The Authors should revise the references from the first paragraph.... For example: the statement about hematotoxic potential of SVMP, SVSP and PLA2 is already a scientific consensus. Therefore, there are many sources specially from the seminal studies that corroborate with this question. However, the Authors present references from venom proteomic analysis of other snakes to support this question. On the other hand, the authors present coherency in the literature proposed as reference to address the mechanism of action of P-III metalloproteinase. I suggest that his paragraph must be reworded.

Line 208: The presence of class P-II metalloproteinase, which possesses metalloproteinase and disintegrin-like domians was also observed.????
Line 303: The combination of SVMP and SVSP observed in O. monticola venom is responsible for prey attack, incapacitation, and digestion.???

**Response:** We thank the reviewer for comments on discussion. We rewrote discussion by updating and improving literature coherence.

**Reviewer #2**:

It is an interesting work to be published in PLOS. This article describes describe proteomics and immuno characterization of snake *Ovophis monticola* venom. I have the following comments that the authors may like to consider:
Line 95
- Provide the total number of individuals, age, sex, locality (adults or young people, males, females)
- It is important to provide some functional analysis

**Response:** Biological and geographical information regarding all snakes is provided as a table in materials and methods (Table 1; page 5 line 105). In this particular study, we focused mainly on providing the inventory of all protein constituents of Asian mountain pit viper *O. monticola* venom. Those with antigenic properties reacted with monovalent and hematotoxic polyvalent antivenom were given. However, we did not perform the experiments to analyse the functions.

Discussion:
Despite little published literature on *Ovophis monticola*:
Please, include in the discussion this reference:
Pandey, D. P., Chaudhary, B., & Ram Shrestha, B. (2021). Documentation of a proven Mountain Pitviper (*Ovophis monticola*) envenomation in Kathmandu, Nepal, with its distribution ranges: implications for prevention and control of pitviper bites in Asia. Journal of venom research, 11, 1–6.
Mainly about clinical characteristic of poisoning by this specie and lack of specific treatment.
Provide conclusion or future perspectives in the last paragraphy about immunocharacterization results/ envenomation treatment.
Line 527
Exclude the number 75.

**Response:** We cited the above literature as a reference in a revised version of manuscript (ref no.5).
