# Supplementary material for: Proteomics and immunocharacterization of Asian mountain pit viper (Ovophis monticola) venom
Source: PLoS One. 2021 Dec 1;16(12):e0260496. doi: 10.1371/journal.pone.0260496 (PMC8635378; doi:10.1371/journal.pone.0260496)
Supplement: Attachment — Submitted filename: Response to reviewers.docx [file pone.0260496.s005.docx]

PONE-D-21-18686R1

Proteomics and immunocharacterization of Asian Mountain pit viper (Ovophis monticola) venom

PLOS ONE

**Additional Editor Comments (if provided):**

Based on the new round of peer-round some minor concerns have arisen. I recommend a minor revision before acceptance of the manuscript.

**Reviewers' comments:**

**Reviewer #1:** The authors rewrote the manuscript and provide relevant references and endeavored to comply with all referee requests. I just emphasize that only Ovophis don't belong to a taxonomic system. This task dont were revide for authors. However. The authors present a study with high quality in methodology, data generation and data analysis, producing significant contribution for toxinology field. Thus, this work is suitable for publication on PLOS ONE.

**Response:** We corrected *Ovophis* to *Ovophis* spp. as suggested by the reviewer (e.g. lines 68, 83, 91, 288, 305, 313, 396)

**Reviewer #3:** The article highlights the spectrometry-based proteomics and the immunoreactivity of the venom of the *Ovophis monticola* viper. It is a work based on an excellent premise and the methodologies are very well defined. I recommend the work for publication. However, some minor revisions are needed.

1. In the session "Two-dimensional polyacrylamide gel electrophoresis (2DE)" (Material and Methods), the authors state that a 100 µg sample of venom was used. However, since the protein concentrations were measured using the Lowry method, please review whether these 100 µg are venom or protein.

**Response: :** It was 100 μg of protein. We corrected the sentence in line 135.

2. In the session “Proteomic analysis of *O. monticola* venom” (Results), the first paragraph deals with a methodological aspect.

**Response:** We rewrote this paragraph according to the reviewer’s suggestion.

3. The same observation applies to the following topics. Instead of repeating the concepts of the methodology, the objective could be presented if evaluating that result, since this information is not present in the text.

**Response:** We revised result session and eliminated repetition particularly on methodology.

4. In “Immunoreactivity of protein antigens in *O. monticola* venom to monovalent and polyvalent antivenoms by indirect ELISA”, the information “Since there is no homospecific antivenom to Ovophis venoms currently available, all pit viper envenoming victims usually receive either monovalent antivenom (raised against *T. albolabris* venom) or hematotoxic polyvalent antivenom (produced against venoms *of C. rhodostoma, D. siamensis and T. albolabris*) to alleviate symptoms” is applicable to the discussion, requiring references.

**Response:** We clarified this point by providing a reference (line 232, ref no. 16 )

5. Figures and tables should be self-explanatory. In the case of table 2, a legend stating the meaning of MW, PI, emPI must be provided.

**Response**: We added the explanation in the table 2 legend (lines 238-241, 244-246) and the definition of abbreviation is provided as in a legend as suggested.

6. Discussion: What is the toxinological consequence related to the fact that the venom has more acidic than basic protein spots? How does this interfere with symptoms related to accident? Is this positive or negative regarding the immunoreactivity of the venom, especially with the polyvalent serum? Present references.

**Response:** In this revised version of manuscript, we discussed the above points raised by the reviewer with relevant references, lines 323-327, 391-395. In addition, to make table 4 more readable, we added a column of MW of non-immunoreactive peptides.

7. Why are some references in numerical format (ex (22) (23)) and others are in nominal format (ex Damm et al, 2021)?

**Response:** We corrected all references appeared in text in numerical format according to Plos guideline.
